# Investigation of Impact Strength and Hardness of UHMW Polyethylene Composites Reinforced with Nano-Hydroxyapatite Particles Fabricated by Friction Stir Processing

**DOI:** 10.3390/polym11061041

**Published:** 2019-06-12

**Authors:** Imran Khan, Ghulam Hussain, Khalid A Al-Ghamdi, Rehan Umer

**Affiliations:** 1 Department of Mechanical Engineering, University of Engineering and Technology, Peshawar 25120, Pakistan; engrimran@uetpeshawar.edu.pk; 2 Faculty of Mechanical Engineering, GIK Institute of Engineering Sciences and Technology, Topi 23640, Pakistan; 3 Department of Industrial Engineering, King Abdulaziz University, Jeddah 21589, Saudi Arabia; kaaalghamdi@kau.edu.sa; 4 Department of Aerospace Engineering, Khalifa University of Science and Technology, Abu Dhabi 127788, UAE; rehan.umer@ku.ac.ae

**Keywords:** friction stir processing (FSP), polymer composites, impact strength, Rockwell hardness, nano-particles

## Abstract

The impact strength and surface properties of polymeric materials are of critical importance in various engineering applications. Friction stir processing (FSP) is a novel method for the fabrication of composite materials with superior mechanical properties. The main objective of this study is to investigate the impact strength and Rockwell hardness of UHMW polyethylene composites reinforced with nano-hydroxyapatite particles fabricated through FSP. The spindle speed (*ω*), tool traverse speed (*f*), volume fraction (*v*) of strengthening material and shoulder temperature (*T*) were key processing parameters. The analysis of variance (ANOVA) indicated that the selected processing parameters were significant. Microscopic investigations unveiled that high levels of (*v, f*) and low levels of (*T, ω*) caused agglomeration of the reinforcing particles and induced voids and channels, which consequently reduced the impact strength and hardness of the manufactured composite. However, medium conditions of processing parameters exhibited better distribution of particles with minimum defects, and hence resulted in better mechanical properties. Finally, the models to predict the impact strength and hardness are proposed and verified. Sets of process parameters favorable to maximize the impact strength and Rockwell hardness were worked out, which were believed to increase the impact strength, Rockwell hardness number, and ultimate tensile strength by 27.3%, 5.7%, and 11.2%, respectively.

## 1. Introduction

Polymer matrix composites have been widely used in various industries such as aerospace, automotive, and marine industries. The mechanical properties, including impact and hardness, are critically important for these applications. These properties are strongly dependent on the composition of the material and the fabrication process. Polymers alone usually do not fulfill high strength requirements for many of these applications. Hence, numerous research studies have focused on the modification of polymeric matrices without altering their bulk properties.

The ultra-high molecular weight polyethylene (UHMWPE) is an exceptional class of polyethylene (PE) with an average molecular weight 10 times that of conventional high density polyethylene (HDPE) resins. The applications include conveyor bands, sprockets, cradles, cores of golf balls, ski and snow board surfaces, and noise reducing materials. UHMWPE has also found applications in automotive sector such as truck and dump truck bed liners [1]. Adding to that, UHMWPE has many uses in the biomedical engineering applications, especially in joint replacement and implants [1,2]. These implants are utilized in medical apparatuses for orthopedics applications. To date, millions of successful implants have been carried out for hip, shoulder articulating surfaces, and knees [2].

Nano-hydroxyapatite (nHA) has been employed in the biomedical industry for its excellent biocompatibility and bioactivity. When mixed with polymer matrix such as UHMWPE, the resulting composites offer enhanced mechanical and surface properties, along with features of biocompatibility, bioactivity, and anti-bacterial characteristics [3,4,5].

Friction stir processing (FSP) is an emerging technique employed for the production of composites with improved mechanical properties. Initially, Mishra et al. [6] investigated FSP as an adaptation of the friction stir welding (FSW) technique. FSW is a solid-state joining process for metals and was first employed for joining aluminum-based alloys [6]. Various studies have been carried out on the FSW of metals [7,8]. FSP is not considered as a solid-state method for polymeric materials, due to the different melting temperatures of different materials. Conventional FSP tools will create defects such as voids and channels, hence the melting of the polymer material in the nugget zone (NZ) (or stir zone (SZ)) must be performed [9]. The melting in the NZ can be performed by using a specialized tool, commonly known as a ‘shoe-shaped shoulder FSP tool [10].

Recently, there has been an increased interest in FSP and FSW of polymeric materials [11,12,13,14]. In most of these studies, the tensile behavior of the weld zone is investigated. Only a single study was performed focusing on the effects of FSW parameters on impact strength of polymers by Abdel-Gwad et al [15]. Abdel-Gwad et al showed that the impact and tensile strength of the friction stir welded polymeric material (in this case, HDPE) increased with an increase in spindle speed, right up to a certain limit, then lowered with further rise in the spindle speed. Moreover, the tensile strength and impact strength of the friction stir welded polymeric sheet increased with a decrease in the traverse speed, except at the lowest traverse speed. It was also observed that high traverse speeds may lead to milling action instead of joining, and higher spindle speeds may lead to an outpouring of melted sheet, thus reducing the impact and tensile strengths of the composite.

In another study, Azarsa and Mostafapour [12] fabricated polymer-metal nanocomposites via the FSP method, where they observed the agglomeration of nano material at high traverse speeds. Moreover, low shoulder temperatures resulted in several voids and channels, whereas material burning/degradation occurred at high shoulder temperatures. Similarly, Azarsa and Mostafapour [16] studied the effects of FSW parameters on the flexural strength of HDPE sheets and reported that the flexural strength enhanced with rise in spindle speed, and lowered with rise in tool traverse speed. On the other hand, the flexural strength of HDPE sheets increased with an increase in shoulder temperature up to 110 °C, then decreased with further increase in the shoulder temperature (up to 150 °C). Various defects were formed, such as surface cracks and voids, mostly at low spindle speed and low shoulder temperatures, eventually reducing the flexural strength. They also concluded that at higher rotational speeds, considerable material degradation can happen.

To the best of our knowledge, no one investigated the effects of FSP parameters on impact strength of a particle’s reinforced polymer composite. In light of the above views, the main focus of this research is to study the impact strength and hardness behavior of UHMWPE/nHA composites fabricated through the friction stir processing technique. Microscopic and macroscopic analysis reveal the dispersion of strengthening material and various defects. The impact strength and surface hardness of the composites are quantified, and the results are co-related to the microscopic analysis. Empirical models for mechanical properties are developed and verified through experiments. The composite at an optimum set of parameters with reasonably improved mechanical properties and minimum defects are also manufactured, which can prove to be a better candidate material for biomedical and other industrial applications.

## 2. Materials and Methods

### 2.1. Materials

UHMWPE (Ningjin Hongbao Chemical Company Ltd., Shandong, China) and nano Hydroxyapatite (nHA) powder (Xi’an Lyphar Biotech Company Ltd., Xian, China) were used as the matrix and the reinforcement material, respectively. The UHMWPE sheets were cut into rectangular sheets of 200 mm × 165 mm with a thickness of 5 mm. The properties of the parent sheets are given in Table 1. The particle size of nHA powder used in this research was 60 nm (average), having a needle-like shape and 96% purity.

**Figure 1:**
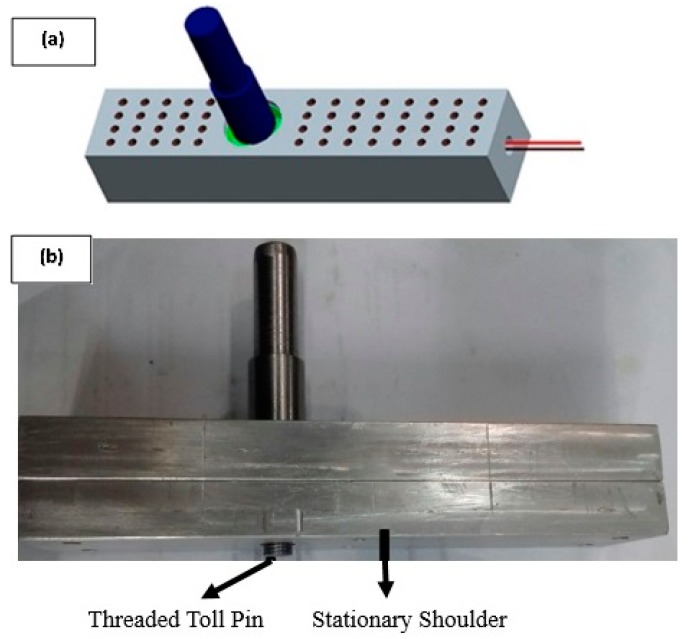
Novel friction stir processing (FSP) tool with holes in the shoe-shaped shoulder (**a**) Schematic and (**b**) Actual tool used.

The FSW/FSP tools are not considered suitable for processing polymers as compared to metals [17]. For attaining desired mechanical properties, a novel tooling setup has been employed here. In this particular setup, the step of closing the upper surface of the polymer sheet to avoid the outpouring of strengthening particles is eliminated, which eventually decreases the production cost and time. The tooling employed in this study is shown in Figure 1.

### 2.2. Experimental Setup

In this tooling setup, a thrust bearing was installed, allowing the tool pin to rotate relative to the stationary shoulder, as shown in Figure 1. Moreover, 5 mm diameter holes were drilled on the upper surface of the shoulder, as this helped in reducing the time delay for achieving the desired shoulder temperature. A quick response time (average 4 s relative to the shoulder without holes) was achieved. The lower surface of the tool was well finished and coated with PTFE to avoid any sticking that might occur between the aluminum hot shoulder and melted surface of the polymer. The tool pin and stationary shoulder were made from hot-worked steel and 7075 aluminum, respectively. To control the shoulder temperature and to provide external heating, a closed-loop heating system was utilized. A cartridge heater (length of 80 mm and power of 400 Watt) was also employed to heat the shoulder.

Before commencing the process, a channel of specific dimensions was formed in the polymer sheet to adjust the strengthening material, followed by compression of the powder in the channel. The volume percentage (*v*) of the strengthening material can be calculated by the formula given in Equation (1), i.e., “the sectional area (*Ac*) of the channel divided by the total processed area (*At*) multiplied by 100”. The volume percentage (*v*) of strengthening material was altered by varying the dimensions of the channel. The dimension of the channel for *v* of 5, 10, and 15% were (160 × 1 × 2), (160 × 2 × 2), and (160 × 3 × 2) mm^3^, respectively.

(1) $$v=\;\frac{Ac}{At}\;\times \;100$$

Before setting-up the range of processing parameters, it was noticed that the tool shoulder temperature (*T*) of more than 100 °C was found to be very high, and was closer to the melting temperature of UHMWPE [13,18]. The room temperature (which in this case was 30 °C) was selected as the lower limit, and 65 and 100 °C were the other two levels of *T*. The maximum limit of volume percentage (*v*) of the reinforcing material in the literature [13,19] was found to be 15%, beyond this range, agglomeration and ineffective mixing were observed. The *v* of 5%, 10%, and 15% were chosen as appropriate levels of *v*. We also observed material burning at high spindle speeds (*ω*), e.g., at 2000 rpm. Any spindle speeds lower than 350 rpm may result in ineffective mixing of two materials. Hence, based on our experimentation and literature review, 660, 1200, and 1700 rpm were set as the levels of *ω* [13,19]. Higher tool traverse speed (*f*) means that there is less time for mixing the materials. Hence, machining action might happen at high *f* rather than mixing (when the tool moves quickly it will carry material with itself instead of stirring it just like machining of any material). Therefore, 30, 48, and 85 mm/min were selected as the levels for *f*. Table 2 shows the levels of the processing parameters.

To ensure that the tool shoulder exerts enough pressure on the polymer sheet, and to prevent outpouring of melted polymer, a tool-offset depth of 0.2 mm was used in the plunging step of the process (FSP). Moreover, we define the dwell time as the time required to heat the polymer sheet to produce a puddle of semi-molten material. We noticed that a dwell time higher than 15 s resulted in excessive heating action of the tool pin, resulting in burning of the polymer chains in the nugget zone [20]. Hence, a dwell time of 15 s was employed in the composite manufacturing.

### 2.3. Design of Experiments

Design Expert-10 statistical software package was employed to formulate the experimental test plan. Response surface method (RSM) with I-optimal design was chosen, as I-optimal design reduces the average variance of prediction over the design space, and RSM takes into account the combined effect of parameters along with their sole effects, and requires a lower number of tests [21]. Table 3 shows the test plan that is comprised of twenty-three experiments, including four repeats. The composite fabrication was performed based on this test plan. The friction stirred sheets were allowed to cool down at ambient conditions, while still placed in the fixture to avoid any deformation or shape change.

### 2.4. Mechanical Testing

To eliminate any irregularities from the surface of the friction stirred sheet, face-milling of 0.5 mm was completed on both sides of the sheet. Due to face-milling, the impact strength (I-S) and Rockwell hardness (RH) were affected as −1% to 1.8% and −1.9% to 2.1%, respectively, which are in acceptable ranges. Samples for microscopic analysis, impact, and hardness tests were cut from the processed sheets. The Charpy impact test was performed to determine the I-S of the composite. An impact test was performed according to ISO 179-1/1eB on Shimadzu Charpy impact tester (Shimadzu, Kyoto, Japan). Three tests were performed for each processing condition, and an average value was taken. Rockwell hardness tests were performed using XHR-150 plastic Rockwell hardness tester (shanghai Jinwei Instrument Manufacturing Co. Ltd, Shanghai, China) following ASTM D785 standard. Rockwell E scale was selected to measure the hardness of the composite region of the friction stirred sheets. Hardness values were measured on five different points on the composite material, and an average value was taken. A microscopic analysis was performed by TESCAN scanning electron microscope (TESCAN, Brno, Czech Republic).

## 3. Results

### 3.1. Mechanical Properties of the Composite

The average values from the impact and hardness tests of the composite and parent material are presented in Table 4. The units of I-S and RH are KJ/m**^2^** and hardness Rockwell E-Scale value (HRE). The highest value of the above-mentioned properties have been highlighted in Table 4. The experiments at low (*v, ω,* and *f*) and high *T* (experiment #12) showed a 44% increase in I-S, which is the highest I-S in all the experiments. Moreover, experiment #11 (low ***v*** with medium level of *T, f*, and *ω*) exhibited a 26.5% increase in the I-S. It can be observed that most of the experimental results exhibited an increased I-S, confirming the effectiveness of the process. On the other hand, it can also be observed that the experiment at high *T* and ***v*** and low *ω* (experiment #7) showed that the highest RH was 101 HRE. Experiment #11 (low ***v*** with medium level of *T, f,* and *ω*) showed a 3% increase in the RH.

### 3.2. Analysis of Variance

Analysis of variance (ANOVA) was performed to find out the process parameters which are significant at a 95% confidence level, and the effect on mechanical properties (I-S and RH) of the composite. Table 5 lists the ANOVA results, which proves that the selected processing parameters were significant, either as stand-alone parameters (*f* in case of I-S) or through interaction with other parameters. The order of significance for I-S was *v* > *f > T > ω* and RH was *ω* > *T* > *v* > *f*.

### 3.3. Effects of FSP Parameters on Impact Strength of the Composite

**Figure 2:**
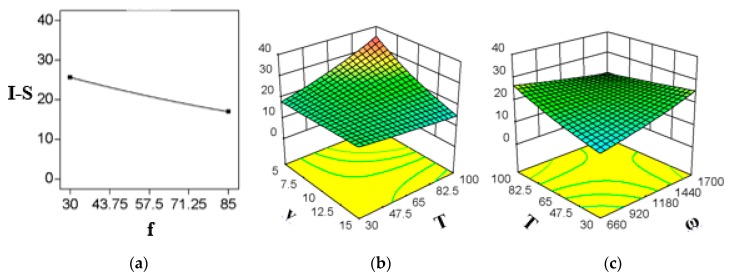
Significant parameters and interactions for composite’s Impact strength (I-S) (**a**) *f* (stand-alone parameter), (**b**) *vT* interaction, and (**c**) *Tω* interaction.

Figure 2 represents the significant interactions and stand-alone parameters for composites impact strength (I-S). Figure 2a represents the effects of *f*, which is a stand-alone parameter in this case. The results show that it does not interact with other parameters. The I-S decreases with an increase in *f*, which differs from the findings by Abdel-Gwad et al [15]. This might be due to less time available for the tool to mix the materials effectively. Figure 2b shows that an increase in *T* causes an increase in I-S at low ***v***, and the same is true for low *ω,* which is obvious from Figure 2c. However, I-S was negligibly effected by *T* at high *v* and it slightly decreased at high *ω,* when *T* was increased at those conditions. Abdel-Gwad et al [15] observed the same effect at which I-S increased with an increase in ***ω*** up to 1200 rpm, then decreased with a further increase in *ω*. The previous studies did not investigate the effect of *T*, as the tool used in that study was not hot-shoe FSP tool. Moreover, Figure 2b shows that by increasing ***v*** the I-S decreases significantly at high *T*. The effect of *ω* is shown in Figure 2c. It can be observed that I-S increases when *ω* is increased at low *T*. However, contradictory results were observed at high *T*.

### 3.4. Effects of FSP Parameters on Hardness of the Composite

**Figure 3:**
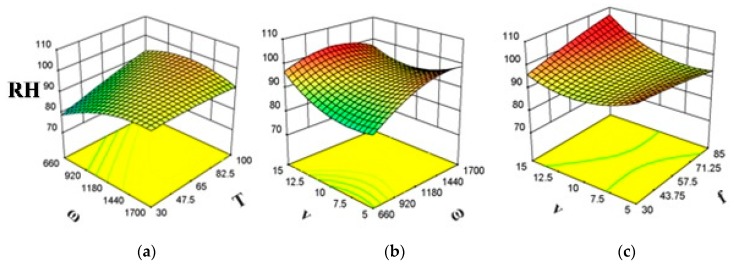
Significant interaction for composite’s RH (**a**) *ωT*, (**b**) *vω*, and (**c**) *vf.*

Figure 3 shows the effect of significant interactions on RH of the fabricated composite. Figure 3b,c shows that RH increases with an increase in *v*, regardless of *f*. The same is true for low *ω*. However, RH first decreases and then increases as *v* is increased at high *ω*. From Figure 3a, it can be observed that RH increases significantly as *T* is increased at low *ω*. However, RH decreased slightly with an increase in *T* at high *ω*. Figure 3c shows that at high *v*, RH increases with an increase in *f.* However, *f* effects RH negligibly at low *v*. Moreover, Figure 3a,b shows that RH increases with an increase in *ω* at low *T* and low *v*. However, at high *T* and high *v*, RH first increases, and then decreases as *ω* is increased.

### 3.5. Empirical Models and Validation

Empirical models are equations of hyper-surfaces which state the relation between the output responses and the chosen processing parameters, and therefore can be employed to predict the responses. The statistical software, Design Expert, predicted the empirical relations for impact strength (I-S) and Rockwell hardness (RH), which are presented in Equations (2) and (3), respectively.

(2) $$\begin{array}{ll} \mathrm{Ln}\left(I-S\right) & =\;0.559\;+\;1.442E-03\;\omega \;-\;2.764E-03\;f\;+\;0.154\;v\;+\;0.033\;T\;\\ & +\;3.834E-06\;\omega f\;-\;6.519E-05\;\omega v\;-\;1.274E-05\;\omega T\;\\ & -5.963E-04\;fv\;-\;3.395E-05\;fT\;-\;1.327E-03\;vT \end{array}$$

(3) $$\begin{array}{ll} \mathrm{Ln}\;\left(\mathrm{RH}\right) & =4.014\;+\;7.467E-04\;\omega \;-2.312E-03\;f\;-\;0.032\;v\;+\;6.105E-03\;T\;\\ & +\;8.282E-07\;\omega f\;-\;1.377E-05\;\omega v\;-\;2.343E-06\;\omega T\;\\ & +\;3.011E-04\;fv\;-\;2.799E-06\;fT\;-1.223E-04\;vT\;-1.828E\\ & -07\;\omega^{2}\;-\;9.642E-06\;f^{2}\;+\;2.203E-03\;v^{2}\;-\;7.008E-06\;T^{2} \end{array}$$

The R^2^ value for each model was above 80% which indicates that the data points follow the model curves, and can be utilized for the prediction of output responses over the entire design space. Moreover, the above models were used to predict the output responses for sets of processing parameters. These results, along with experimental results, were compared for additional validation of the proposed model curve (s). Table 6 lists these conditions which include three sets of processing parameters. These conditions were not included in the test plan. Table 6 confirms that the experimental values are in close agreement with the values predicted by the models; the prediction error ranges from −4.82% to 0.8%. This affirms that the models are reasonably correct.

### 3.6. Microscopic and Macroscopic Analysis

**Figure 4:**
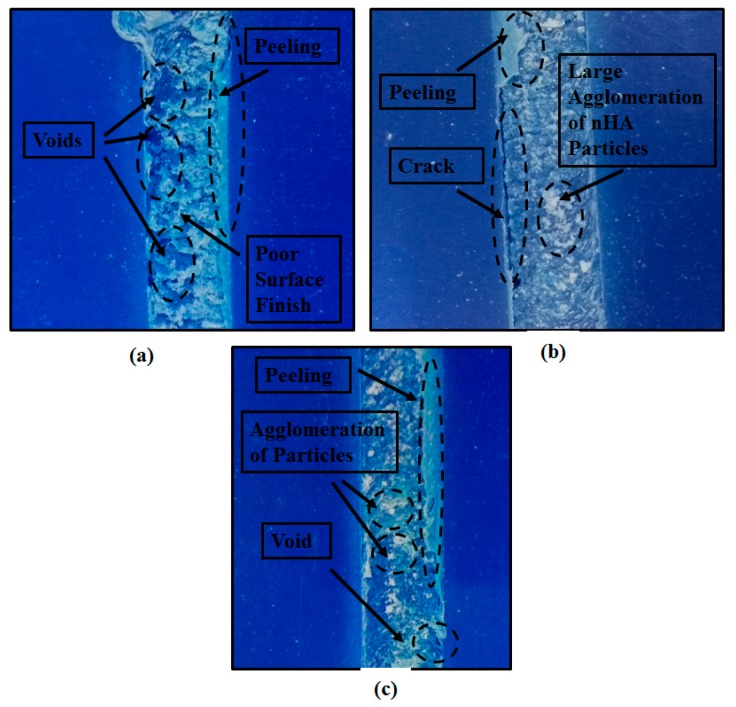
Defects observed in various experiments: (**a**) experiment at low *T* and low *ω*, (**b**) experiment at low *ω*, high *v*, and high *f* and (**c**) experiment at high *v* and high *f*.

Various defects occurred at the conditions which resulted in minimum heat generation and poor mixing, such as high *f*, low *T*, low *ω*, and high *v* or a combination of these parameters. Low *T* means less external heating to melt the polymer chains. Hence, causing poor mixing and various surface defects as observable from Figure 4a and also discussed by Azarsa and Mustafapur [12]. High *f* means that there is less time for the two materials to mix, and hence results in voids and channels (due to machining action) as observable from Figure 4b,c, and discussed by Azarsa and Mustafapur [12] and Abdel-Gwad et al [15]. High *v* means large amount of strengthening material inside the polymer matrix which resulted in agglomeration of the nano particles, and poor mixing as observable from Figure 4b,c. Low *ω* means ineffective mixing and less frictional heat generation between the tool surface and material to be processed. Hence, resulting in large and small channels (as a result of poor bonding between the processed material and the parent material) as observable from Figure 4a,b.

**Figure 5:**
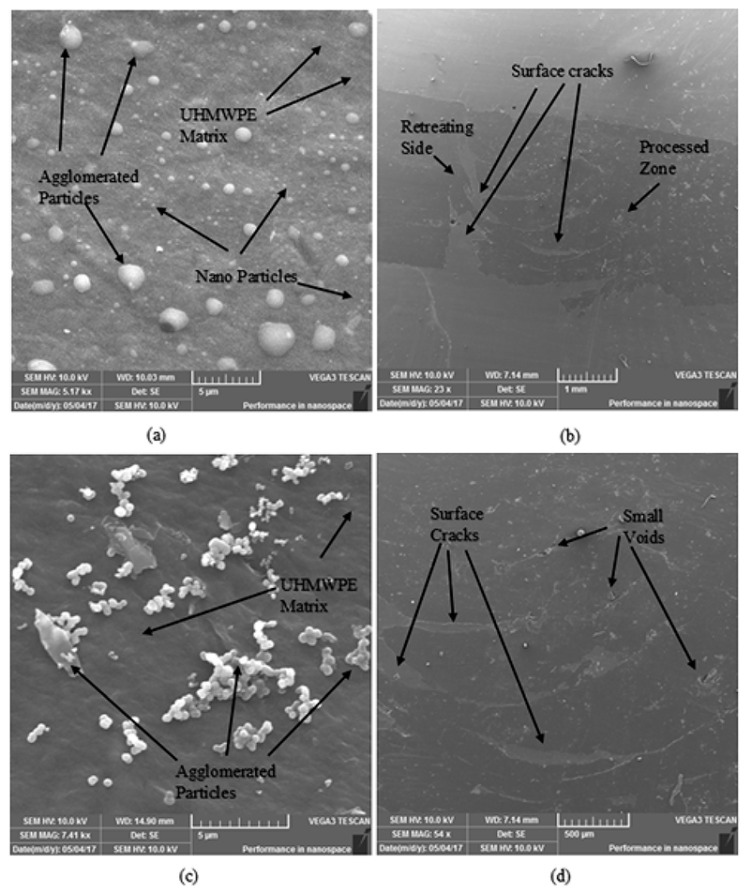
SEM images showing various defects at (**a**) high (*v* and *f*), (**b**) low *ω*, (**c**) high (*f* and *v*), and low *T*, and (**d**) low *ω* high *f.*

Figure 5a,c shows SEM images, confirming agglomeration of the nano powder at high *v* and high *f*. The size of the agglomerated particles is approximately 0.2–2.5 μm. Whereas, the nano powder has an approximate size of 70–95 nm. Figure 5b,d shows SEM images of the composite, when fabricated at low *ω* and high *f*, resulting in surface cracks.

High *f*, low *T*, low *ω*, and high *v* or a combination of these parameters resulted in various defects such as voids, channels, surface cracks, agglomeration, and poor surface finish. These defects, in turn, caused reduction of the I-S and RH as discussed in Section 3.3 and Section 3.4, respectively. Hence, these parameters are not recommended for the fabrication of the composite.

**Figure 6:**
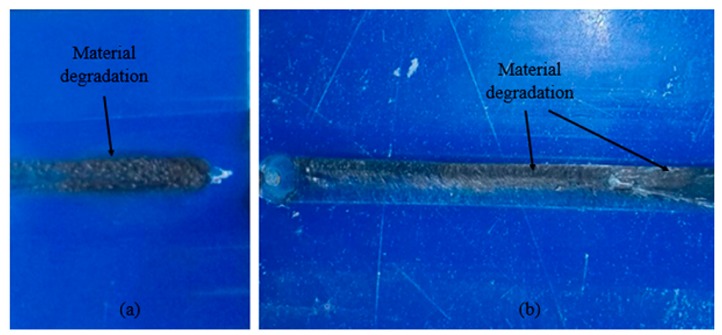
Material degradation in: (**a**) experiment 4 (at high *T* and high *ω*) and (**b**) experiment 6 (at high (*T* and *ω*) and low *f*).

Moreover, material burning has been observed in experiments with combinations such as high *T* and high *ω*, high *T* and low *f*, high *ω* and low *f*, as noticeable from Figure 6 (change of blue color into brown). The combinations of processing parameters which generate high heat, resulted in material burning/degradation, as discussed by Azarsa and Mostafapour [12,16]. This material burning might affect the bulk properties of the composite, i.e., biocompatibility and bioactivity, etc. Hence, these combinations of process parameters must be avoided in the fabrication of the composite. On the contrary, the combinations which resulted in material burning showed high impact strength, as discussed in Section 3.3. This might be due to the high stirring action of the FSP tool (high *T* with low *f* or high *ω* results in relatively more stirring time and high frictional heat), resulting in the strengthening of the composite, but at the same time, affects its biocompatibility.

**Figure 7:**
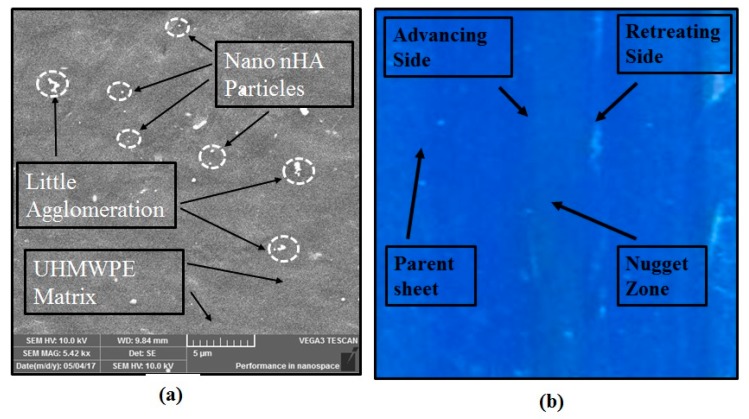
Experiments with better results: (**a**) Relatively better nano hydroxyapatite (nHA) particle distribution in experiment 11, and (**b**) Better surface finish in experiment 23.

The experiments at medium conditions of three processing parameters, such as high *T* with medium levels of *ω*, *f* and *v*, and low *v* with medium levels of *ω*, *f* and *T* resulted in improved surface finish, and is observable from Figure 7. It was observed that these processing parameters are suitable for better material mixing, and therefore resulted in an improved surface finish. Moreover, material burning did not occur at these conditions. Better particle distribution can be seen at these combinations, as shown in Figure 7a. Due to an improved distribution of strengthening particles and minimum defects, the experiments at these processing parameters show an enhancement in mechanical properties, as observable from Table 4.

### 3.7. Optimum Conditions

From the above discussion, it is clear that the following conditions should be avoided in the fabrication of UHMWPE/nHA composite in order to increase the hardness and I-S of the composite, and to avoid material degradation, voids, and agglomeration:

*ω* = (600 & 1700) rpm, *v* = 15%, *f* = (30 & 85) mm/min, *T* = (30 & 100) °C

Table 7 presents optimum set of process parameters which were suggested by using the software. This combination showed a 27.2% increase in impact strength, and a 5.7% increase in hardness through mechanical tests. Similarly, the ultimate tensile strength (UTS) and % elongation were also measured to be 20.7 MPa and 8.17 mm/mm, respectively, i.e., 11.2% increase in UTS with negligible change in % elongation as compared to parent material. Moreover, material degradation did not occur at these conditions. Hence, this set of parameters is suggested for the fabrication of composites through FSP.

The optimum conditions proposed in the above table were validated by conducting experiments at the proposed conditions. The results from these experiments were in good agreement with those reported in the above table. Hence, the proposed parameter conditions can serve as reliable guidelines for the improved impact strength and Rockwell hardness of UHMWPE/nHA composite fabricated through FSP techniques.

## 4. Conclusions

In this study, friction stir processing of UHMWPE/nHA polymer nanocomposite was successfully performed. The fabrication was completed by varying a number of processing parameters which included spindle speed (*ω*), volume fraction of nHA (*v*), tool traverse speed (*f*), and shoulder temperature (*T*). The microstructural analysis was performed to further investigate and verify the results. The significant conclusions from this investigation are as follows; all processing parameters were found significant (either as stand-alone parameters or in interaction with the other parameters) for impact strength (I-S) and Rockwell hardness (RH). Various defects were observed at low *ω* (660 rpm), low *T* (30 °C), and high *v* (15%), which include voids, surface cracks, channels, and agglomeration. These defects reduced the I-S and RH of the fabricated composite. Material burning was observed at processing conditions of low *f* (30 mm/min) and low *v* of nHA particles (5%) with high *ω* (1700 rpm) and high *T* (100 °C). Material burning/degradation can affect the inherent properties, such as biocompatibility of the material, hence these conditions must be avoided in the fabrication of the composite. High nHA content (15%) and high *T* (100 °C) resulted in high RH, i.e., 7.5% harder than the parent material. Low *f* (30 mm/min) with low *v* of nHA content (5%) and high *T* (100 °C) resulted in a 44% increase in I-S as compared to the parent material. The following conditions should be avoided in the fabrication of UHMWPE/nHA composite in order to increase the I-S and RH of the composite, and to avoid material degradation, voids, and agglomeration:

*ω* = (600 & 1700) rpm, *v* = 15%, *f* = (30 & 85) mm/min, *T* = (30 & 100) °C

The range of processing parameters, i.e., *ω* of 1200 rpm, *f* of 48 mm/min, *T* of 65 °C with 5–10% *v* resulted in higher I-S and RH. The following optimum conditions were proposed to fabricate the polymer composite based on UHMWPE matrix via FSP technique, which is believed to increase the I-S, RH, and ultimate tensile strength by 27.3%, 5.7%, and 11.2%, respectively, with respect to the parent material:

*T* = 75 °C, *v* = 5%, *f* = 48 mm/min and *ω* = 1200 rpm.

## Figures and Tables

**Table 1:** Properties of the Base Material.

Property	Value and Units	Symbol
**Ultimate tensile strength**	18.6 MPa	UTS
**Tensile modulus**	160 MPa	E
**Strain at failure**	8.3 mm/mm	e _failure_
**Impact strength (u-notched)**	23 KJ/m^2^	I-S
**Rockwell hardness**	94 HRE	RH
**Melting temperature range**	130–138 °C	MP
**Nature**	semi crystalline	-
**Density**	0.958 Kg/m^3^	-
**Size**	500 mm × 500 mm × 5 mm	-

**Table 2:** Processing parameters and their respective levels.

Parameters	Symbols	Units	Levels		
			−1	0	+1
**Spindle Speed**	*ω*	rpm	660	1200	1700
**Traverse Speed**	*f*	mm/min	30	48	85
**Volume Fraction**	*v*	%	5	10	15
**Shoulder Temperature**	*T*	°C	30	65	100

**Table 3:** Experimental test plan.

Exp. No.	*ω*	*f*	*v*	*T*
**1**	660	30	15	65
**2**	1700	30	5	30
**3**	660	48	5	65
**4**	1700	85	15	100
**5**	660	85	15	30
**6**	1700	30	10	100
**7**	660	48	15	100
**8**	1700	85	10	30
**9**	660	30	10	100
**10**	1700	48	15	30
**11**	1200	48	5	65
**12**	660	30	5	100
**13**	660	85	10	100
**14**	1700	85	5	100
**15**	1200	48	5	65
**16**	1700	48	10	65
**17**	660	85	5	30
**18**	660	48	10	30
**19**	1200	48	10	100
**20**	1700	85	15	100
**21**	1200	30	15	30
**22**	1700	48	10	65
**23**	1200	48	10	100

**Table 4:** Mechanical properties of the friction stir processed composite.

Exp. No.	I-S	Relative I-S	RH	Relative RH
	KJ/m^2^	%	HRE	%
**1**	21.50	93.48	89.10	94.79
**2**	28.46	123.75	97.60	103.83
**3**	25.61	111.34	92.30	98.19
**4**	8.50	36.96	**100.40**	**106.81**
**5**	11.00	47.83	95.00	101.06
**6**	32.00	139.13	90.00	95.74
**7**	22.00	95.65	101.00	107.45
**8**	30.32	131.85	94.80	100.85
**9**	29.84	129.75	96.00	102.13
**10**	20.00	86.96	95.80	101.91
**11**	29.10	126.52	96.80	102.98
**12**	**33.12**	**144.00**	93.00	98.94
**13**	21.06	91.55	90.33	96.10
**14**	31.00	134.78	97.60	103.83
**15**	29.10	126.52	97.50	103.72
**16**	14.93	64.92	90.60	96.38
**17**	8.00	34.78	72.00	76.60
**18**	12.50	54.35	77.00	81.91
**19**	25.54	111.03	94.10	100.11
**20**	8.00	34.78	97.20	103.40
**21**	29.50	128.26	96.00	102.13
**22**	14.93	64.92	92.00	97.87
**23**	25.54	111.03	97.10	103.30
**Base Material**	23.00	100.00	94.00	100.00

**Table 5:** Analysis of variance (ANOVA) Results.

Source	RH		I-S	
	*p*-Value	Significance (Yes/No)	*p*-Value	Significance (Yes/No)
**Model**	Quadr. * (0.01)	Y	**2FI** (0.01)	Y
***ω***	0.01	Y	0.19	N
***f***	0.43	N	0.05	Y
***v***	0.05	Y	0.01	Y
***T***	0.01	Y	0.13	N
***ωf***	0.38	N	0.52	N
***ωv***	0.02	Y	0.07	N
***ωT***	0.01	Y	0.01	Y
***fv***	0.02	Y	0.39	N
***fT***	0.84	N	0.70	N
***vT***	0.15	N	0.02	Y
***ω^2^***	0.06	N	-	-
***f^2^***	0.78	N	-	-
***v^2^***	0.02	Y	-	-
***T^2^***	0.72	N	-	-

* Quadratic model.

**Table 6:** Comparison between the experimental and predicted responses values.

S. No.	Process Parameters				I-S (KJ/m^2^)			HR (HRE)		
	*ω*	*f*	*v*	*T*	Pred ^1^	Exp ^2^	% Error	Pred	Exp	% Error
**1**	1200	48	5	45	21.53	21.8	−1.25	94.39	94.2	0.02
**2**	660	85	7.5	30	10.14	10.05	0.8	74.6	78.2	−4.82
**3**	660	85	13	45	12.26	12.51	−2.03	89.02	92	−3.3

^1^ Predicted and ^2^ Expected.

**Table 7:** Optimum conditions to maximize impact strength and Rockwell hardness.

Scenario	Processing Parameters				Mechanical Properties		Desirability
	*ω*	*f*	*v*	*T*	I-S (KJ/m^2^)	RH (HRE)	
**1 (generic)**	1200	48	5	75	29.2	99.35	0.89
**2 (from test plan)**	1200	48	5	65	26.5	97.80	0.8
